# Intact brain processing of musical emotions in autism spectrum disorder, but more cognitive load and arousal in happy vs. sad music

**DOI:** 10.3389/fnins.2014.00192

**Published:** 2014-07-15

**Authors:** Line Gebauer, Joshua Skewes, Gitte Westphael, Pamela Heaton, Peter Vuust

**Affiliations:** ^1^Music in the Brain, Department of Clinical Medicine, Center of Functionally Integrative Neuroscience, Aarhus UniversityAarhus, Denmark; ^2^Interacting Minds Centre, Aarhus UniversityAarhus, Denmark; ^3^Department of Psychology, Goldsmiths, University of LondonLondon, UK; ^4^The Royal Academy of MusicAarhus, Denmark

**Keywords:** autism spectrum disorder, music, emotion, fMRI

## Abstract

Music is a potent source for eliciting emotions, but not everybody experience emotions in the same way. Individuals with autism spectrum disorder (ASD) show difficulties with social and emotional cognition. Impairments in emotion recognition are widely studied in ASD, and have been associated with atypical brain activation in response to emotional expressions in faces and speech. Whether these impairments and atypical brain responses generalize to other domains, such as emotional processing of music, is less clear. Using functional magnetic resonance imaging, we investigated neural correlates of emotion recognition in music in high-functioning adults with ASD and neurotypical adults. Both groups engaged similar neural networks during processing of emotional music, and individuals with ASD rated emotional music comparable to the group of neurotypical individuals. However, in the ASD group, increased activity in response to happy compared to sad music was observed in dorsolateral prefrontal regions and in the rolandic operculum/insula, and we propose that this reflects increased cognitive processing and physiological arousal in response to emotional musical stimuli in this group.

## Introduction

Music is highly emotional; it communicates emotions and synchronizes emotions between people (Huron, 2006; Overy, 2012). The social-emotional nature of music is often proposed as an argument for why music has sustained such prominence in human culture (Huron, 2001; Fitch, 2005). Indeed, people spend quite a large amount of their time listening to music. A recent Danish survey found that 79% of people between 12 and 76 years listened to music more than 1 h daily (Moesgaard, 2010), and when people are asked why they listen to music they consistently say that it is because music induces and regulates emotions (Dubé and Le Bel, 2003; Rentfrow and Gosling, 2003). Processing of emotional music is found to engage limbic and paralimbic brain areas, including regions related to reward processing (for reviews see Koelsch, 2010; Peretz, 2010; Zald and Zatorre, 2011). However, not everybody experience and process emotions in the same way. For instance, people with autism spectrum disorder (ASD) are often found to be impaired in recognizing, understanding and expressing emotions (Hobson, 2005).

ASD is a complex neurodevelopmental disorder characterized by difficulties in social and interpersonal communication, combined with stereotyped and repetitive behaviors and interests (APA, 2013). Despite somewhat conflicting findings, studies indicate that people with ASD have difficulties identifying emotions from facial expressions (Boucher and Lewis, 1992; Celani et al., 1999; Baron-Cohen et al., 2000; Philip et al., 2010; see however Jemel et al., 2006), affective speech (Lindner and Rosén, 2006; Golan et al., 2007; Mazefsky and Oswald, 2007; Philip et al., 2010; see however Jones et al., 2011), non-verbal vocal expressions (Hobson, 1986; Heaton et al., 2012) and body movements (Hubert et al., 2007; Hadjikhani et al., 2009; Philip et al., 2010). These difficulties in emotion recognition are associated with altered brain activations in people with ASD compared to neurotypical (NT) controls, i.e., with less activation in the fusiform gyrus and amygdala when viewing emotional faces (Critchley et al., 2000; Schultz et al., 2000; Ashwin et al., 2007; Corbett et al., 2009), and abnormal activation of superior temporal gyrus (STG)/sulcus and inferior frontal gyrus when listening to speech (Gervais et al., 2004; Wang et al., 2006; Eigsti et al., 2012; Eyler et al., 2012). Accordingly, it has been suggested that people with ASD do not automatically direct their attention to emotional cues in their surroundings, but instead tend to perceive emotions more analytically (Jemel et al., 2006; Nuske et al., 2013).

It has previously been advocated that general social-emotional difficulties could make people with ASD less emotionally affected by music and less able to recognize emotions expressed in music (Huron, 2001; Levitin, 2006). Nonetheless, anecdotal reports dating all the way back to Kanner's (1943) first descriptions of autism seems to suggest quite the opposite, namely, that people with autism enjoy listening to music, become emotionally affected by music, and often are musically talented. Behavioral studies have shown that people with ASD process musical contour and intervals just as well as NT individuals (Heaton, 2005), and that they display superior pitch processing (Bonnel et al., 1999; Heaton et al., 1999; Heaton, 2003) and pitch memory (Heaton, 2003; Stanutz et al., 2014). Interestingly, behavioral studies have shown that children and adults with ASD correctly identify a wide range of emotions in music just as well as NT individuals (Heaton et al., 2008; Allen et al., 2009a,b; Caria et al., 2011; Quintin et al., 2011). A qualitative study by Allen et al. (2009b) found that adults with ASD listened to music as often as people without ASD, and when asked why they listened to music, they reported being emotionally affected by the music and feeling a sense of belonging to a particular musical culture. Moreover, Allen et al. (2013) recently showed that physiological responses to music are intact in people with ASD, despite a lower verbal responsiveness to music in this group.

Only one brain imaging study of processing of musical emotions in ASD has been published to this date. Caria et al. (2011) scanned 8 adults with Asperger's syndrome (AS) while listening to excerpts of classical and self-chosen musical pieces. Emotion ratings of valence and arousal showed no difference between the two groups. Their main neurobiological finding was significant activations of a variety of cortical and subcortical brain regions, including bilateral STG, cerebellum, inferior frontal cortex, insula, putamen and caudate nucleus, in response to emotional music, which were common for the ASD and NT group. Yet, between-group comparisons revealed less brain activation in individuals with AS relative to NT individuals in response to both happy and sad music. For happy music, AS individuals showed decreased brain activation relative to NT individuals in the right precentral gyrus, supplementary motor area and cerebellum. When comparing self-chosen favorite happy music with standard happy music the ASD group showed decreased brain responses in supplementary motor area, insula and inferior frontal gyrus compared to the NT group. For sad music, individuals with AS showed decreased brain activation in precentral gyrus, insula and inferior frontal gyrus. Taken together, Caria et al. (2011) concludes that the most prominent difference between the two groups is the decreased activation of left insula in individuals with AS relative to NT individuals during processing of emotional music. This difference might be explained by higher levels of alexithymia (Fitzgerald and Bellgrove, 2006; Bird et al., 2010), an inability to identify and describe feelings, in the AS group compared to the NT group. This study is important in that it is the first to directly investigate the neural processing of emotional music in individuals with ASD. However, the study is limited by a fairly small sample size and it is not apparent if the ASD and the NT group were matched on IQ and/or verbal IQ. Previous studies have shown that differences in emotion processing might depend on verbal IQ rather than having ASD as such (Lindner and Rosén, 2006; Golan et al., 2007; Heaton et al., 2008; Anderson et al., 2010). Besides, the stimuli used by Caria et al. (2011) included a mix of familiar and unfamiliar music (5 familiar/self-chosen and 5 unfamiliar excerpts of happy and sad music respectively). More studies are needed to generalize these findings to larger groups of people with ASD. Empirical investigations of similarities and differences in the neurocognitive processing of music are relevant for understanding the nature of emotional impairments in individuals with ASD. In the literature on music emotions, the distinction between emotion perception and emotion induction is central (Gabrielsson, 2002; Juslin and Laukka, 2004; Konečni, 2008). The present study focused on emotion perception from music in individuals with ASD, because this study was designed to act as a parallel to ASD studies on emotion recognition in other domains (facial expressions, affective prosody, body movements etc.). Thus, the aim of the present study was to investigate emotion recognition and neural processing of happy, sad and neutral music in high-functioning adults with ASD compared to a group of NT individuals matched on age, gender, full-scale IQ and verbal IQ.

## Materials and methods

### Participants

A total of 43 participants were included in the study, 23 of these had a formal diagnosis of ASD. Participants with ASD were recruited through the national autism and Asperger's association, assisted living services for young people with ASD, and specialized educational facilities. The structural MRI of three participants with ASD showed abnormal ventricular enlargement (this is not an uncommon finding see Gillberg and Coleman, 1996) and were excluded before data analysis was begun. One ASD participant was unable to relax in the scanner and thus did not complete the testing. Consequently, a total of 19 high-functioning adults with ASD (2 females, 17 males) and 20 NT adults (2 females, 18 males) were included in the data analysis.

All participants were right-handed and native speakers of Danish, with normal hearing. Groups were matched on gender, age, IQ, and verbal IQ (Table 1). All participants were IQ-tested using Wechsler's Adult Intelligence Scale (WAIS-III, Wechsler, 1997). None of the NT participants had any history of neurological or psychiatric illness. All participants with ASD carried a previous formal diagnosis of ASD. Diagnoses were supported by the autism diagnostic observation schedule (ADOS-G, Lord et al., 2000) at the time of the study. All participants with ASD were invited back in for the ADOS testing after the brain scanning session, but unfortunately five participants were unable to come back for testing due to long transportation, or because they needed special assistance. Thus, 14 out of the 19 ASD participants completed ADOS testing (Table 2). Nonetheless specialized psychiatrists had previously diagnosed all participants with ASD, and we were given access to their medical records to further confirm diagnoses. All ASD participants were medication naïve at the time of the study, and did not have any comorbid psychiatric disorders. All participants gave written informed consent and were compensated for their time and transportation expenses. The study was approved by the local ethics committee and was in accordance with the Helsinki declaration.

**Table 1:** **Participant characteristics**.

	**ASD**	**NT**	***t*-value *p*-value**
Age (*SD*/range)	26.16 (5.63/20–36)	24.45 (4.57/19–41)	1.04 *ns*
Gender	2 F/17 M	2 F/18 M	−0.05 *ns*
Full-scale IQ^a^(*SD*/range)	108.32 (14.56/78–135)	114.50 (12.37/92–137)	−1.43 *ns*
Verbal IQ^b^ (*SD*/range)	112.68 (23.7/74–186)	118.30 (13.8/90–143)	−1.05 *ns*
MET^c^ melodic (*SD*/range)	35.47 (6.22/24–49)	37.10 (5.05/28–50)	−0.90 *ns*
MET^c^ rhythmic (*SD*/range)	36.79 (5.67/24–45)	37.35 (4.43/28–48)	−0.32 *ns*

a WAIS-III full-scale IQ (Wechsler, 1997).

b Verbal subscale of WAIS-III.

c The Musical Ear Test (Wallentin et al., 2010). ns, not significant at p < 0.05.

**Table 2:** **ADOS scores for ASD participants (N = 14)**.

ADOS total (*SD*/range)	11.2 (4.5/3–18)
Communication (*SD*/range)	3.5 (1.7/1–6)
Social interaction (*SD*/range)	7.2 (3.9/1–12)
Imagination and creativity (*SD*/range)	0.33 (0.5/0–1)
Stereotyped and repetitive behaviors (*SD*/range)	1.6 (1.8/0–6)

Scores from the Autism Diagnostic Observation Schedule (ADOS, module 4, Lord et al., 2000) for 14 out of the 19 ASD participants. The remaining 5 ASD participants did not complete ADOS testing, see participant information for further information on this.

### Measures of musical experience

All participants completed a musical background questionnaire asking about their musical preferences, musical training, listening habits, and general physiological and emotional responses to music. For questions about physiological and emotional responses to music, participants rated how much they agreed (from 1 to 5, where 1 is least, 5 is most) with statements like “I find it easy to recognize whether a melody is happy or sad” or “I can feel my body responding physically when I listen to music.” To compare musical abilities between the two groups, participants completed the Musical Ear Test (Wallentin et al., 2010), which measures melodic and rhythmic competence on a same/different listening task.

### Stimuli

Emotional stimuli (happy/sad) were instrumental excerpts of 12 s duration, taken from the beginning of real music pieces of different genres (see Appendix A for a complete list of musical excerpts). Emotional stimuli were selected from a corpus of 120 musical excerpts based on pilot-data from a separate group of 12 neurotypical adults. The 120 musical excerpts were rated for emotionality by the pilot-group on a 5-point Likert-scale ranging from very sad to very happy. A total of 40 music excerpts (20 happy/20 sad) were selected for the fMRI experiment. The 20 happy excerpts, selected for the study, were all rated as happy or very happy by all participants in the pilot group. Similarly, the 20 sad excerpts were rated sad or very sad. During the piloting of the stimuli, people were asked whether they were familiar with the music, and if they could name the artist or part of the title of the piece. If any of the pilot-participants could name artist of part of the title of the musical piece from which the excerpt was taken, the excerpt was not included in the final stimulus sample for the fMRI-experiment. Besides the 20 happy and the 20 sad music excerpts, a 12 s chromatic scale was used as a neutral control condition. The neutral control condition acted as an “auditory baseline” for the two emotion conditions. This was done to have some degree of auditory stimulation across all conditions, while only the emotional intensity varies. Stimuli were all matched on duration and volume.

### Design

**Figure 1:**
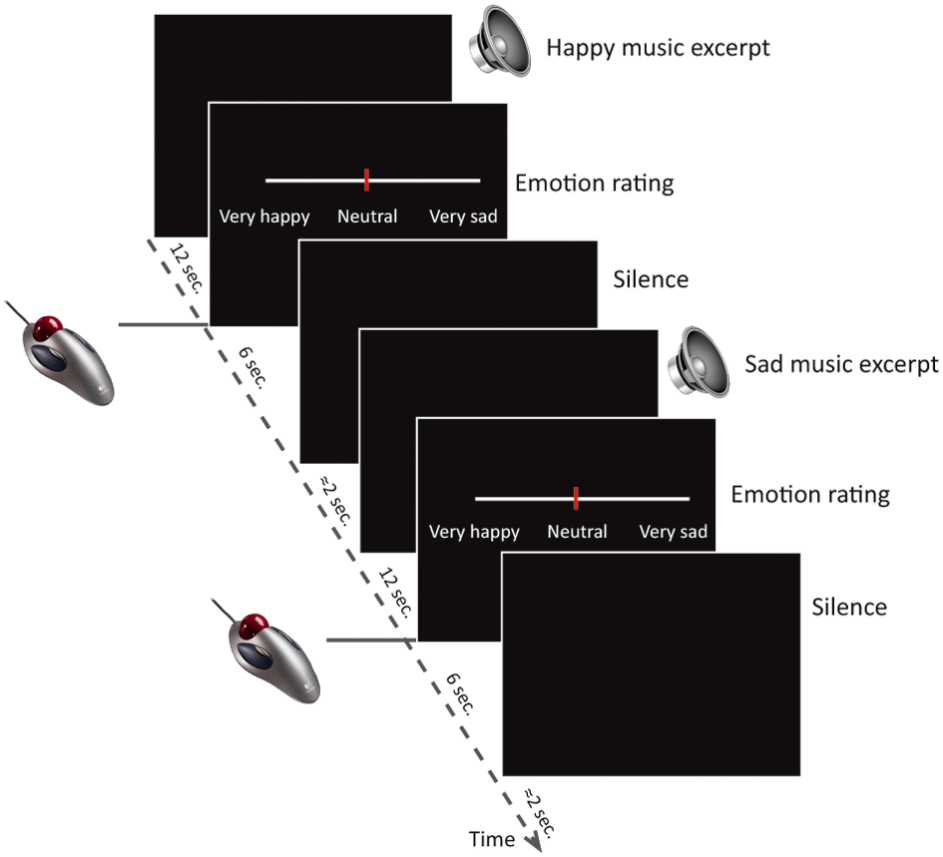
**Study design.** The study consisted of 60 trials (20 happy, 20 sad, 20 neutral) inside the MR scanner. Each trial consisted of a musical excerpt of 12 s duration, followed a visual analog scale depicted on the screen in front of the participant for 6 s while participants indicated their emotion intensity ratings by using an MR-compatible scroll-ball mouse. After the rating there were approximately 2 s silence before the next trial began.

Participants listened to the music excerpts and rated the perceived emotion inside the fMRI-scanner. The study used a block-design, where 20 trials of happy instrumental music, 20 trials of sad and 20 trials of neutral music were presented in pseudo-random order. After hearing each excerpt (12 s), participants had 6 s to rate the perceived emotional intensity of the music on a visual analog scale from very sad over neutral to very happy (Figure 1). The visual analog scale was displayed on an MR compatible screen in the center of the participant's visual field and ratings were given with an MR compatible scroll ball. Participants were instructed that neutral was right in the middle, and the cursor always started out in the neutral position. No visual feedback was displayed during the music listening, but participants were instructed to lie with their eyes open during the entire scan. All participants completed 5 trials outside the scanner, to make sure that they were familiar with the task and understood the instructions. It was emphasized that it was the emotion expressed in the music, and not the emotion or pleasantness experienced by the participant in response to the music, that should be rated. Participants also completed a similar task while listening to emotional linguistic stimuli. The data from this task will be analyzed and published separately of the present study.

### MRI acquisition

Brain imaging was obtained using a Siemens, 3T Trim Trio, whole-body magnetic resonance scanner located at the Centre of Functionally Integrative Neuroscience at Aarhus University Hospital, Denmark. Two 10,5 min experimental EPI-sequences were acquired with 200 volumes per session and the parameters; *TR* = 3000 ms, *TE* = 27 ms, flip angle = 90°, voxel size = 2.00 × 2.00 × 2.00 mm, #voxels = 96 × 96 × 55, slice thickness 2 mm, no gaps. Participants wore MR-compatible headphones inside a 12-channel head coil, and had a trackball in their right hand for the valence ratings. After the functional scans a sagittal T1-weighted anatomical scan with the parameters; *TR* = 1900 ms, *TE* = 2.52, flip angle = 9°, voxel size = 0.98 × 0.98 × 1 mm, # voxels = 256 × 256 × 176, slice thickness 1 mm, no gaps, 176 slices, was acquired for later co-registration with the functional data. Participants were instructed to lie still and avoid movement during the scan.

### Behavioral data analysis

Age, gender, IQ, musicianship and answers on the background questionnaire were compared between groups using independent samples *t*-test. Emotion ratings were analyzed using a 2 (groups) × 3 (emotion condition: happy, neutral, or sad) mixed model analysis of variance (ANOVA).

### fMRI analysis

fMRI data analysis was performed using Statistical Parametric Mapping (SPM8 version 4667; http://www.fil.ion.ucl.ac.uk/spm) (Penny et al., 2011). Preprocessing was done using default settings in SPM8. The functional images of each participant were motion corrected and realigned, spatially normalized to MNI space using the SPM EPI template and trilinear interpolation (Ashburner and Friston, 1999), and smoothed using an 8 mm full-width at half-maximum smoothing kernel.

For each participant, condition effects were estimated according to the general linear model (Friston et al., 1994). To identify clusters of significant activity across the two groups, one-sample t-contrasts for the main effect of emotional vs. neutral prosody were performed across all participants. For between group differences, random-effects analyses were performed using independent-samples *t*-tests. All results are thresholded at *p* < 0.01 after family wise error correction (FWE, Friston et al., 1996) with an extent threshold at 10 voxels. *p* < 0.01 after FWE correction is a relatively conservative significance threshold, thus to avoid type-2 errors all between-group analyses were also done with a threshold of *p* < 0.05 after FWE correction. Figures are t-statistics displayed on top of standard MNI T1-images. Labeling of brain regions is done according the Wake Forest University (WFU) PickAtlas (Lancaster et al., 2000; Tzourio-Mazoyer et al., 2002; Maldjian et al., 2003). However, the WFU PickAtlas does not label midbrain structures very precisely, so for identifying activity in ventral striatum and nucleus accumbens we used a midbrain atlas specialized for the basal ganglia (Ahsan et al., 2007). Tables indicate coordinates for peak-voxels significant at both peak and cluster-level.

## Results

### Behavioral results

No statistically significant group differences were found with regard to gender, age, full-scale IQ or verbal IQ (Table 1). We found no group difference with regard to musicianship *t*_(37)_ = −0.85, *p* = 0.403, or musical abilities as measured with the Musical Ear Test (Table 1). On the ‘musical background’ questionnaire, 11 out of the 19 ASD participants, compared to 5 out of the 20 NT participants, reported that they experienced that specific tones had a great influence or special significance to them (i.e., were perceived as especially annoying or particularly pleasant) *t*_(37)_ = 2.157, *p* = 0.038. There were no statistically significant group differences to the questions regarding emotional impact and recognition (“I get emotionally affected by music”; “I find it easy to recognize whether a melody is happy or sad”; “when I am feeling down I often like to listen to sad music”; “It makes me happy to listen to happy music”; “If I am sad, it cheers me up to listen to happy music”). Nor did we see any differences on questions relating to physical arousal associated with music (“It energizes me to listen to music”; “I can feel my body responding physically when I listen to music”; “I often get chills when I listen to music”).

### Emotion ratings

**Figure 2:**
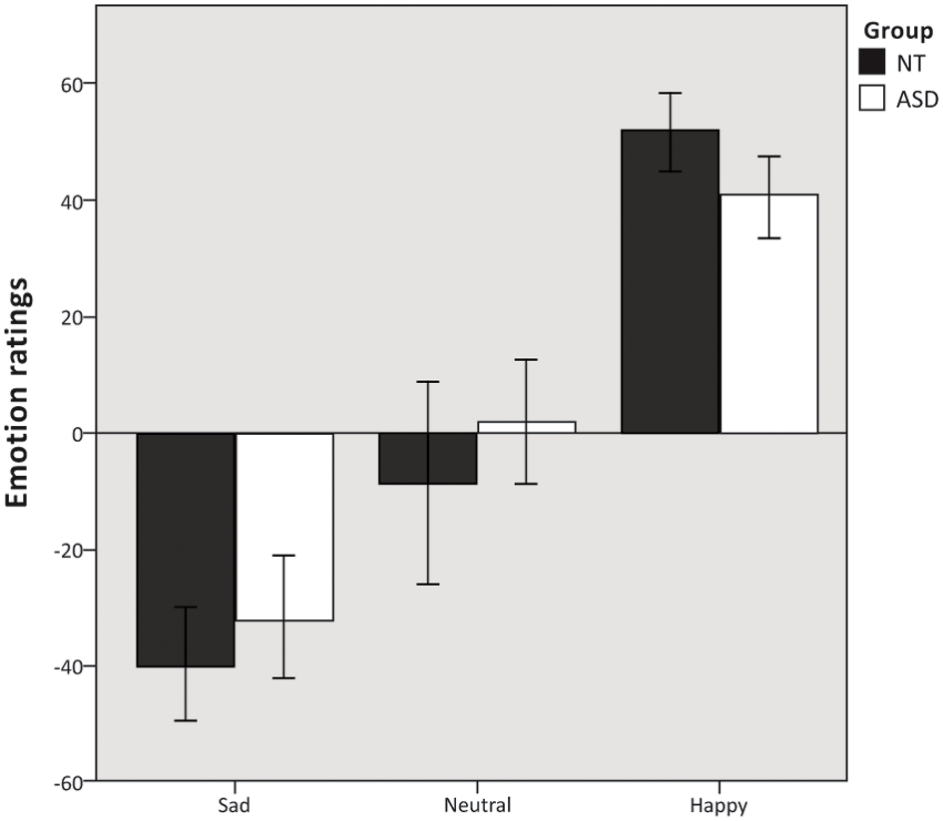
**Emotion ratings.** Mean emotion ratings (on a visual analog scale from −100 to 100) of sad, neutral and happy musical excerpts. Error bars indicate 95% confidence intervals.

Mixed model ANOVA revealed a significant main effect of emotion condition *F*_(2, 37)_ = 120.19, *p* < 0.000. The ANOVA revealed no main effect of group *F*_(1, 37)_ = 0.365, *p* = 0.550 (Figure 2) and no significant group × emotion condition interaction *F*_(2, 37)_ = 2.5, *p* = 0.091. Additional independent samples *t*-test revealed no difference in the number of missing responses between the two groups *t*_(37)_ = −0.928, *p* = 0.359.

### fMRI results

**Figure 3:**
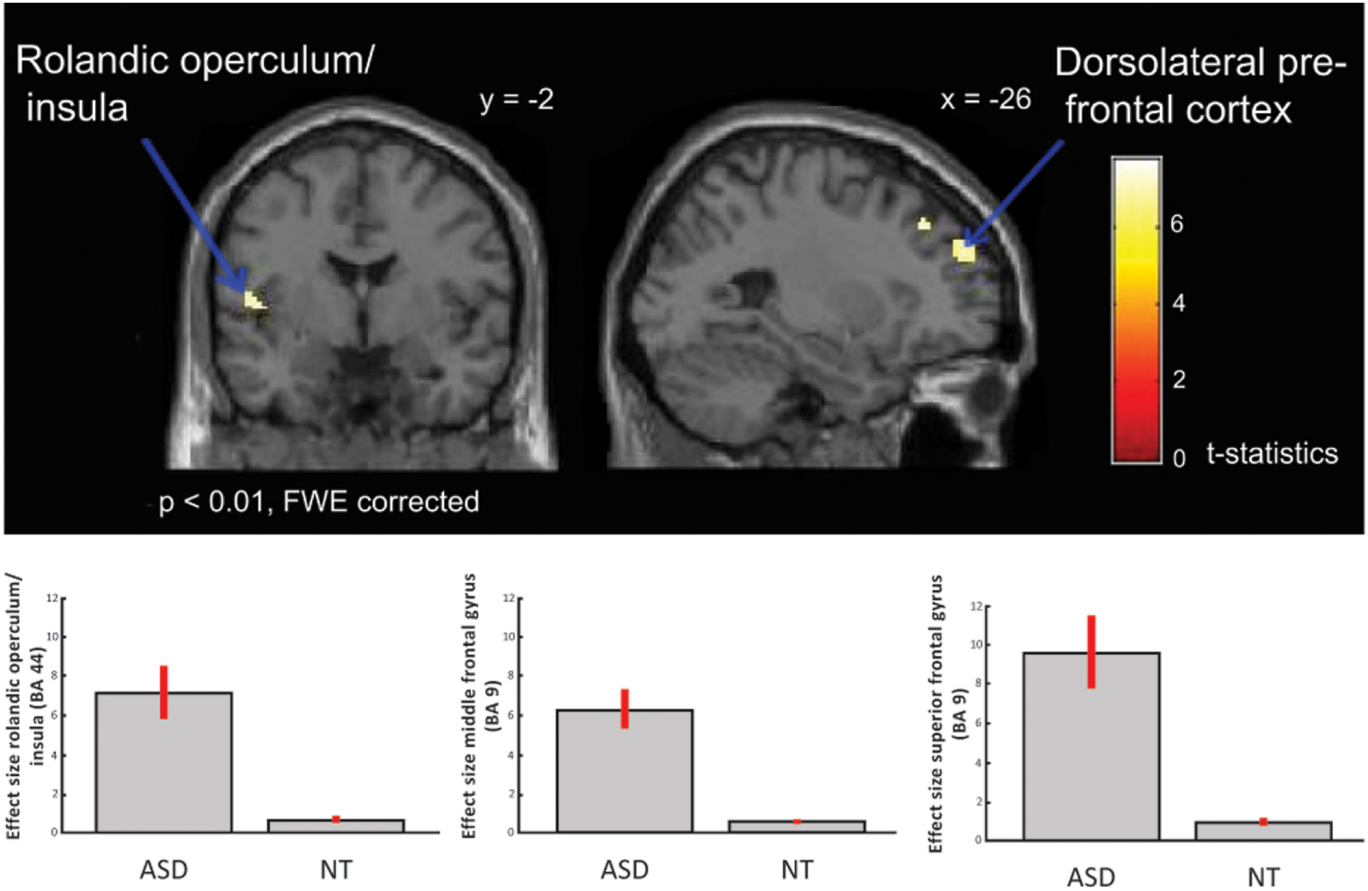
**ASD > NT: group difference for happy vs. sad music (FWE *p* < 0.01).** Individuals with ASD showed increased activation in dorsolateral prefrontal cortex, i.e., middle and superior frontal gyrus, and in insula/rolandic operculum. Box plots show mean effect size for each group in the peak voxel for each region, with 95% confidence intervals.

Between-group comparisons showed no differences on the contrasts; emotional (happy and sad) music vs. neutral music, happy vs. neutral music or sad vs. neutral music at *p* < 0.01 after FWE-correction, nor at a less conservative significance threshold of *p* < 0.05 FWE-corrected. However, the ASD group showed significantly greater activation in response to happy compared to sad music in left dorsolateral prefrontal regions i.e., middle frontal gyrus [*x* = −24, *y* = 34, *z* = 42; *T*_(1, 38)_ = 7.75; BA: 9], left rolandic operculum/insula [*x* = −50, *y* = 2, *z* = 8; *T*_(1, 38)_ = 7.41; BA: 6] and in superior frontal gyrus [*x* = −26, *y* = 52, *z* = 32; *T*_(38)_ = 7.23; BA: 9] (Table 3 and Figure 3), than did the NT group.

**Table 3:** **ASD > NT: group difference for happy vs. sad music (FWE *p* < 0.01)**.

		**BA**	***x***	***y***	***z***	***k***	***T***
Middle frontal gyrus	L	9	−24	34	42	27	7.75
Rorlandic operculum/insula	L	44	−50	−2	8	27	7.41
Superior frontal gyrus	L	9	−26	52	32	58	7.23

Peak coordinates from significant clusters after FWE correction at the peak level, *p* < 0.01, extent threshold = 10 voxels (Friston et al., 1996).

**Figure 4:**
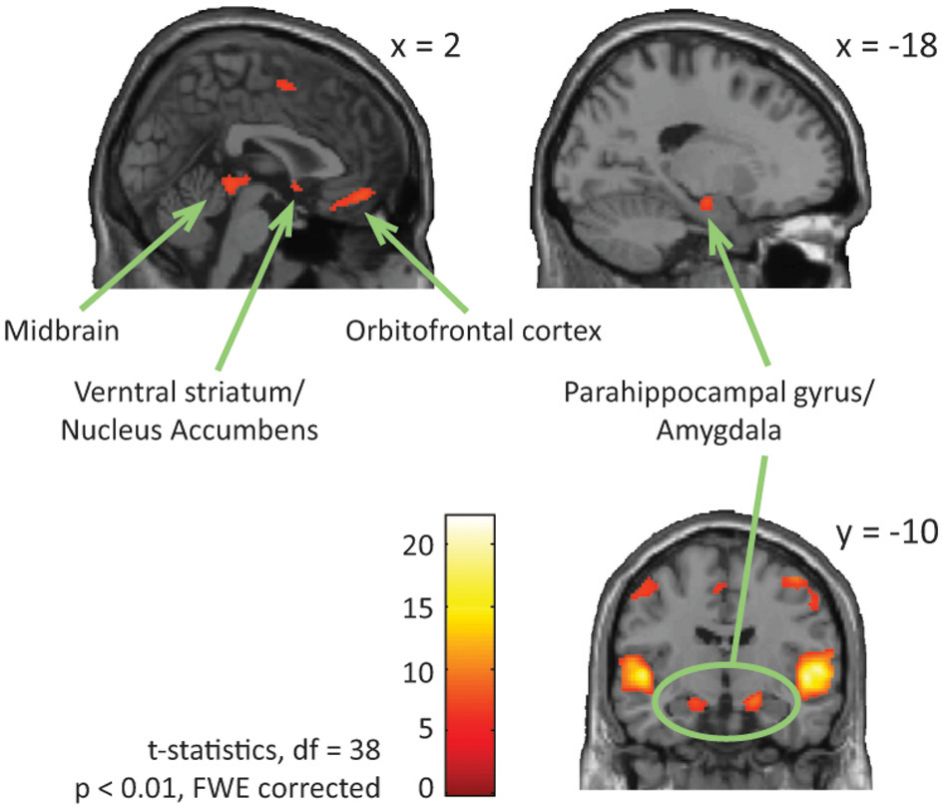
**ASD and NT—main effect of emotional vs. neutral music.** The figure shows brain activations in response to emotional compared to neutral music across all participants, including activations in midbrain, parahippocampal gyrus extending into amygdala, ventral striatum/nucleus accumbens, and orbitofrontal cortex.

Looking at both groups together, we found significant brain activation in response to emotional (happy and sad) music compared to neutral music in; bilateral STG [BA: 22], precentral gyrus [BA: 6, 4], parahippocampal gyrus [BA: 34, 28], left medial orbitofrontal gyrus [BA: 11], left midbrain, bilateral inferior frontal gyrus [BA: 47], right medial frontal gyrus [BA: 6], right ventral striatum/nucleus accumbens, and in orbitofrontal cortex [BA: 11] (Table 4, Figure 4).

**Table 4:** **Main effect of emotional vs. neutral music (FWE *p* < 0.01)**.

		**BA**	***x***	***y***	***z***	***k***	***T***
Superior temporal gyrus	R	22	54	−14	6	3864	22.24
Superior temporal gyrus	L	22	−48	−16	4	2795	18.97
Precentral gyrus	R	6	54	−4	46	285	10.78
Medial frontal gyrus	L	11	−6	42	−14	314	9.50
Midbrain	L		−6	−24	−6	298	8.46
Inferior frontal gyrus	L	47	−26	28	−14	59	8.36
Precentral gyrus	L	4	−42	−18	54	400	8.22
Medial frontal gyrus	R	6	10	0	58	344	8.14
Parahippocampal gyrus	L	28	−18	−10	−16	57	7.49
Ventral striatum	R	25	2	6	−6	17	6.93
Inferior frontal gyrus	R	47	46	28	−4	92	6.88
Inferior frontal gyrus	L	47	−36	26	2	10	6.57
Inferior orbitofrontal gyrus	R	11	38	34	−16	11	6.44

Peak coordinates from significant clusters after FWE correction at the peak level, *p* < 0.01, extent threshold = 10 voxels (Friston et al., 1996).

Comparing processing of happy and sad music across groups showed increased activation bilaterally in STG [BA: 22, 38], anterior cingulate and cingulate cortex [BA: 32, 31], subcallosal gyrus, midbrain, medial frontal gyrus [BA: 9], and postcentral gyrus [BA: 3] (Table 5). No brain regions showed more activity in response to sad than happy music across groups.

**Table 5:** **Main effect of happy vs. sad music (FWE *p* < 0.01)**.

		**BA**	***x***	***y***	***z***	***k***	***T***
Superior temporal gyrus	L	22	−56	−10	2	2566	16.03
Superior temporal gyrus	R	22	56	−12	4	3275	14.54
Middle frontal gyrus	L	9	−24	34	42	256	9.11
Anterior cingulate cortex	L	32	−8	46	0	398	8.15
Subcallosal gyrus	L	11	−14	24	−10	24	7.96
Extra nuclear	R	-	24	2	−10	97	7.90
Superior temporal gyrus	R	38	36	10	−22	43	6.96
Medial frontal gyrus	L	9	−4	50	26	25	6.63
Postcentral gyrus	L	3	−52	−10	52	15	6.55
Superior temporal gyrus	L	22	−58	−60	16	10	6.51
Cingulate	-	31	0	−22	42	11	6.32

Peak coordinates from significant clusters after FWE correction at the peak level, *p* < 0.01, extent threshold = 10 voxels (Friston et al., 1996).

## Discussion

Our results demonstrate intact emotion recognition, and mostly intact neural processing of emotional music in high-functioning adults with ASD compared to NT adults. Across both ASD and NT individuals we found increased activation in limbic and paralimbic brain areas, such as parahippocampal gyrus extending into amygdala, and midbrain structures, also including reward regions, such as medial orbitofrontal cortex and ventral striatum. These regions are highly interconnected and have previously been identified as core regions for emotional processing of music (Koelsch, 2010, see Figure 4), and for other emotional stimuli (Adolphs, 2002). Meanwhile, individuals with ASD displayed significantly greater activation in left dorsolateral prefrontal cortex (i.e., middle and superior frontal gyrus), and left rolandic operculum/insula, in response to happy contrasted with sad music, compared to NT individuals (Figure 3).

The difference in brain activation in response to happy compared to sad music between the two groups could be interpreted as heightened arousal and increased reliance on cognitive processing for emotion recognition of happy music in individuals with ASD. The dorsolateral prefrontal region is associated with higher cognitive functions, such as working memory and executive functions (Boisgueheneuc et al., 2006). With regard to emotion processing the medial parts of the pre-frontal cortex has been found to be involved in emotion appraisal (Etkin et al., 2011). Hence, the increased activation of the dorsolateral prefrontal cortex found in this study, is likely related to a more cognitively demanding emotion recognition strategy for happy music in the ASD group. This would be consistent with the findings of more analytical and cognitive strategies which have been suggested to govern face perception in individuals with ASD (Jemel et al., 2006), and with findings of atypicalities in verbal reporting of emotions (Heaton et al., 2012; Bird and Cook, 2013), including musical emotions in people with ASD (Allen et al., 2009a, 2013).

Meanwhile the insula is critically involved in mediating cognitive and emotional processing, for instance in emotion monitoring and regulation (Menon and Uddin, 2010; Gasquoine, 2014). The insula is highly connected with limbic, sensory and motor regions of the brain, and is considered a central in sensorimotor, visceral, interoceptive processing, homeostatic/allostatic functions, and emotional awareness of self and others (Craig, 2002; Critchley, 2005). Furthermore, the insula is posited to be involved in monitoring emotional salience (Craig, 2009). Consistent with this the insula is previously found to be involved in emotional responses to music (Blood and Zatorre, 2001; Brown et al., 2004; Griffiths et al., 2004; Trost et al., 2012). Hence activation of insula associated with emotional music found in these studies might be due to increased physiological arousal. Indeed, happy music is often found to be more arousing than sad music (see for instance, Trost et al., 2012). Thus the stronger insula activation found in the ASD group in this study might be linked to enhanced bodily arousal in response to happy music compared to NT individuals. This corresponds well with self-reports of individuals with ASD describing stronger physiological responses to music (Allen et al., 2009a).

Interestingly, in our study, we only found evidence for this differential brain processing between individuals with ASD and NT individuals in response to happy compared to sad music. Future studies are needed to clarify whether recruitment of extra cognitive resources is unique to the happy-sad differentiation or is related to other music emotions too.

While subtle brain processing differences were found between the two groups in recognizing happy music, we found no evidence of differences in emotion ratings between the ASD and NT group (Figure 2). The successful emotion recognition seen from our behavioral ratings was further substantiated by the data from the “music background”-questionnaire, where the ASD group reported that they got emotionally affected by listening to music, found it easy to recognize emotions from music, and experienced physiological arousal comparable to that of NT individuals when listening to music in everyday settings. This is consistent with previous findings of intact emotion recognition (Heaton et al., 2008; Quintin et al., 2011), and intact physiological responses to music in high-functioning individuals with ASD (Caria et al., 2011; Allen et al., 2013). Physiological arousal during music listening is associated with emotional and pleasurable responses (Grewe et al., 2005; Salimpoor et al., 2009). Thus, the presence of typical physiological responses to music indicates that people with ASD are not only capable of correctly recognizing emotions in music, but that they also experience full-fledged emotions from listening to music—though they might use an extra effort to report them. In summary our study shows that individuals with ASD do not have deficits in emotion recognition from music in general, but in certain instances rely on partially different strategies for decoding emotions from music, which may result in subtle differences in brain processing.

Looking at brain responses to emotional compared with neutral music we did not find any differences between the two groups. Across all participants we found increased activation in bilateral STG, parahippocampal gyrus extending into amygdala, inferior frontal gyrus, precentral gyrus, left midbrain, and right ventral striatum. This is consistent with what is generally found in studies of music emotion processing (Koelsch et al., 2006; Peretz, 2010; Brattico et al., 2011; Trost et al., 2012; Park et al., 2013). Indeed, music with a high impact on arousal, such as joyful music is associated with increased activation of the STG (Koelsch et al., 2006, 2013; Mitterschiffthaler et al., 2007; Brattico et al., 2011; Mueller et al., 2011; Trost et al., 2012), which also corresponds to our finding of increased STG activation in response to happy compared to sad music. Besides, being engaged in auditory processing, the STG is also central for social and emotional processing (Zilbovicius et al., 2006), and is proposed to code communicative and emotional significance from all social stimuli (Redcay, 2008). Music is a rich tool for communicating emotions (Huron, 2006; Juslin and Västfjäll, 2008), and accordingly emotional music will elicit more activation of the STG compared to neutral music, as was the case in our study. Also, the precentral and inferior frontal gyrus, were more active during emotional music than neutral. Precentral activity is found to correlate with the arousal dimension of music (Trost et al., 2012), and arousal levels are generally found to correlate with emotional responses to music (Grewe et al., 2005; Salimpoor et al., 2009). Increased activation of the inferior frontal gyrus has also ben found in response to pleasant compared to scambled music, and is suggested to reflext music syntactic analysis (Koelsch et al., 2006).

We found increased activation of the parahippocampal gyrus extending into amygdala in response to emotional music across all individuals. Engagement of the parahippocampal gyrus and amygdala are primarily found to respond to negative affective states, including varying degrees of musical dissonance (Blood et al., 1999; Koelsch et al., 2006, 2008), but also to positive affective states and happy music (Mitterschiffthaler et al., 2007). In our study activation of the parahippocampal gyrus and amygdala was not significantly greater in response to sad compared to happy music, suggesting that these structures are implicated in processing of both happy and sad music. We found increased brain activation in midbrain structures, overlapping thalamus, in response to emotional music. Thalamus activity is found to correlate with music-induced psychophysiological arousal and pleasurable chills (Blood and Zatorre, 2001), and is central in processing temporal and ordinal complexity (Janata and Grafton, 2003). Indeed, emotional and pleasurable responses to music seem to be associated with optimal levels of complexity (Berlyne, 1971; North and Hargreaves, 1995; Witek et al., 2014), accordingly it makes sense that we find greater activity in these regions during emotional than neutral music.

Emotional music also engaged parts of the right ventral striatum, including nucleus accumbens, and medial orbitofrontal cortex, these regions are central parts of the brain's dopaminergic reward system (Gebauer et al., 2012), and have previously been associated with strong emotional and pleasurable responses to music (Blood and Zatorre, 2001; Brown et al., 2004; Menon and Levitin, 2005; Salimpoor et al., 2011, 2013). ASD is previously suggested to be associated with deficient reward processing (Kohls et al., 2013), however, the finding of intact activation of the reward system in response to emotional music suggests that music listening has the same pleasurable and motivational impact on people with ASD as it has on NT individuals.

Despite general agreement between the findings of this study and those of the previous neuroimaging study of emotional music perception in high-functioning adults with AS from Caria et al. (2011), there are some deviations. We found increased activation in the ASD group compared to the NT group in response to happy compared to sad music, and no group differences in processing of emotional compared to neutral music overall. Meanwhile, Caria et al. (2011) found less brain activation in various brain regions including precentral gyrus, cerebellum, supplementary motor area, insula and inferior frontal gyrus response to both happy and sad music in individuals with AS relative to NT individuals. However, the study designs are quite different: First, Caria et al. (2011) employed a relatively small study sample, and only had 5 trials in each condition for some of their comparisons. Second, our study required people to decode the emotional intensity of experimenter-selected music directly inside the scanner, while Caria et al. (2011) had their subjects bring half of the music themselves and had them rate the emotion and arousal of all music before the actual scanning. Consequently, our finding of intact emotion recognition and brain responses to music in individuals with ASD might be facilitated by the explicit nature of our task, where participants were directly instructed to evaluate the perceived emotion from the musical excerpts. Other studies have found that individuals with ASD in general perform better on emotion recognition tasks when given more explicit instructions (for review see Nuske et al., 2013) and show more “normalized” brain activity (Wang et al., 2007). Consequently, the difference in brain activation between this study and that of Caria et al. (2011) might relate to differences between active decoding of emotions from music and passive music listening. Also, the participants in Caria et al.'s (2011) study knew the music in advance, and it is well-established that familiarity influences the way we perceive music (Pereira et al., 2011; Van den Bosch et al., 2013), and may change the emotional experience inside the scanner.

Future studies should investigate differences in implicit and explicit emotion perception in music in ASD individuals, and preferably include more emotions than just happiness and sadness. It would also be interesting to conduct similar experiments using alternative scanning protocols such as sparse temporal sampling or interleaved steady state imaging, which might optimize signal intensity from auditory and subcortical regions compared to continuous scanning (Mueller et al., 2011; Perrachione and Ghosh, 2013). Finally, future studies should aim to investigate emotional responses to music in low-functioning and non-verbal individuals with ASD, since these might be the ones who could benefit the most from using music to communicate and share emotions.

## Conclusion

Individuals with ASD showed intact emotion recognition from music, as expressed in their behavioral ratings, and in typical brain processing of emotional music overall, with activation of limbic and paralimbic areas, including reward regions. However, in response to happy compared to sad music individuals with ASD had increased activation of left dorsolateral prefrontal regions and rolandic operculum/insula, suggesting a more cognitively demanding strategy for decoding happy music, and potentially higher levels of physiological arousal in individuals with ASD.

### Conflict of interest statement

The authors declare that the research was conducted in the absence of any commercial or financial relationships that could be construed as a potential conflict of interest.
