# Supplementary material for: Intact brain processing of musical emotions in autism spectrum disorder, but more cognitive load and arousal in happy vs. sad music
Source: Front Neurosci. 2014 Jul 15;8:192. doi: 10.3389/fnins.2014.00192 (PMC4098021; doi:10.3389/fnins.2014.00192)
Supplement: Supplementary file 1 [file DataSheet1.DOCX]

| **Appendix A: Musical excerpts are taken from the following pieces** | |
| --- | --- |
| **Happy music** | |
| **Title:** | **Artist:** |
| Apply some pressure | Mark Ronson ft. Paul Smith |
| Blind | Hercules and Love Affair |
| Diversion | Mark Ronson |
| Gamma Ray | Beck |
| Happy up here | Röyksopp |
| Lights | Scissor sisters |
| Má Vlast: Vltava (Extract), B. 111 | Paavo Berglund & Staatskapelle Dresden |
| Piano Concerto No. 19 in F major, KV 459 - Allegro assai | Jiri Tomasek, Christiane Engel & The Stern Orchestra |
| Narcissist | The Libertines |
| Oh my god | Mark Ronson Ft. Lily Allen |
| Schubert symphony No. 5 B-dur D. 485 - Allegro | London Festival Orchestra - Schubert |
| Sexx laws | Beck |
| Solskin på Nørreport station | Rasmus Nøhr |
| Soul man | Sam & Dave |
| Straighten up and fly | Robbie Williams |
| The suburbs | Arcade Fire |
| The boys are back in town | Thin Lizzie |
| Vagabond | Wolfmother |
| Viva la vida | Coldplay |
| You make my dreams | Hall & Oates |
| **Sad music** | |
| **Title:** | **Artist:** |
| Barber: Adagio For Strings, Op. 11 | David Zinman: Baltimore Symphony Orchestra |
| A Beautiful Mind Soundtrack - Main Theme | A Beautiful Mind Soundtrack - Main Theme |
| Brooks was here | Thomas Newman |
| Cynthia | Soap & Skin |
| Everybody's Gotta Learn Sometimes | Beck |
| Forever | Tina Dickow |
| Green Mile Soundtrack - Coffey On The Mile | Thomas Newman |
| King Kong - James Newton Howard - The Venture Departs | James Newton Howard |
| Mad World | Gary Jules |
| Mr Gaunt Pt 1000 | Soap & Skin |
| Sally And Jack [From The Motion Picture Blow Out] (Album Version) | Pino Donaggio |
| Theme from Shindlers List | John Williams |
| The sun | Soap & Skin |
| This love affair | Rufus Wainwright |
| Twisted ways of blues | Didium and the black bonnie picture |
| Vinterbørn | Under byen |
| Vocalise, Op.34 No.14 | Lynn Harrell, Vladimir Ashkenazy |
| When your blackening show | Our broken garden |
| Windows of the world | Scott Walker |
| You are my sister | Antony and The Johnsons |
